# 24. Longitudinal Assessment of Immune Responses to COVID-19 Vaccines in Solid Organ Transplant Recipients

**DOI:** 10.1093/ofid/ofab466.024

**Published:** 2021-12-04

**Authors:** Megan Powell, Amy C Sherman, Julia Klopfer, Michaël Desjardins, Chi-An Cheng, Yasmeen Senussi, Saee Ratnaparkhi, Xhoi Mitre, Monica Feeley, Andres A Avila Paz, Andy J Kim, Henry Rutherford, Jessica Cauley, Austin Kim, Jun Bai Park Chang, Alexis Liakos, Ann E Woolley, David Walt, Lindsey R Baden

**Affiliations:** 1 BWH Division of Infectious Diseases, Boston, Massachusetts; 2 Harvard Medical School/Brigham and Women’s Hospital, Boston, Massachusetts; 3 Brigham and Women's Hospital, Boston, Massachusetts; 4 Brigham and Women’s Hospital, Boston, Massachusetts; 5 Brigham and Women’s Hosptial, Boston, Massachusetts; 6 Brigham & Women’s Hospital, Boston, Massachusetts; 7 Brigham & Women’s Hospital/Dana-Farber Cancer Institute, boston, Massachusetts; 8 Harvard Medical School/Brigham and Women’s Hospital/Wyss Institute, Boston, Massachusetts

## Abstract

**Background:**

mRNA vaccines for coronavirus disease 2019 (COVID-19) illicit strong humoral and cellular responses and have high efficacy for preventing and reducing the risk of severe illness from COVID-19. Since solid organ transplant (SOT) recipients were excluded from the phase 3 trials, the efficacy of the COVID-19 vaccine remains unknown. Understanding the serological responses to COVID vaccines among SOT recipients is essential to better understand vaccine protection for this vulnerable population.

**Methods:**

In this prospective cohort study, a subset of SOT recipients who were part of our center’s larger antibody study were enrolled prior to receipt of two doses of the BNT162b2 (Pfizer, Inc) vaccine for high resolution immunophenotyping. To date, plasma has been collected for 10 participants on the day of their first dose (baseline), day of their second dose, and 28 days post second dose. 23 healthy participants planning to receive either BNT162b2 or mRNA-1273 (ModernaTX, Inc) were also enrolled, providing plasma at the same timepoints. Ultrasensitive single-molecule array (Simoa) assays were used to detect SARS-CoV-2 Spike (S), S1, receptor-binding domain (RBD) and Nucleocapsid (N) IgG antibodies.

**Results:**

Participant demographics and SOT recipient characteristics are summarized in Table 1. Low titers of anti-N IgG at all timepoints indicate no natural infection with COVID-19 during the study (Fig 1A). There were significantly lower magnitudes for anti-S (p< 0.0001), anti-S1 (p< 0.0001), and anti-RBD (p< 0.0001) IgG titers on the day of dose 2 and day 28 post second dose for SOT recipients compared to healthy controls (Fig 1B,C,D). Using the internally validated threshold of anti-S IgG >1.07 based on pre-pandemic controls, only 50% of the SOT sub-cohort responded to vaccine after series completion (Fig 2). There was a positive trend between months from transplant and anti-S IgG titer (Fig 3).

Table 1: Demographics

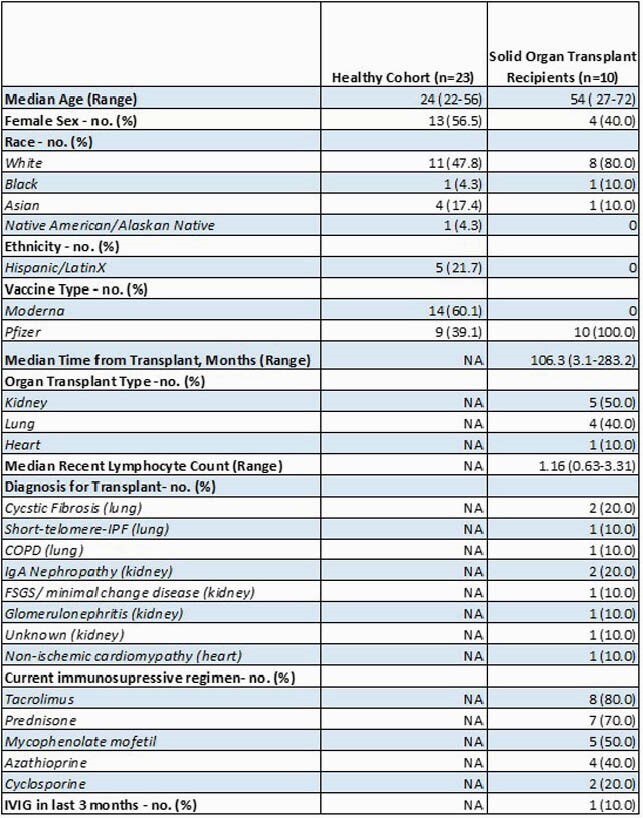

Figure 1: Anti-N, Anti-S, Anti-S1, Anti-RBD and Anti-N Ig G for healthy v. SOT cohort

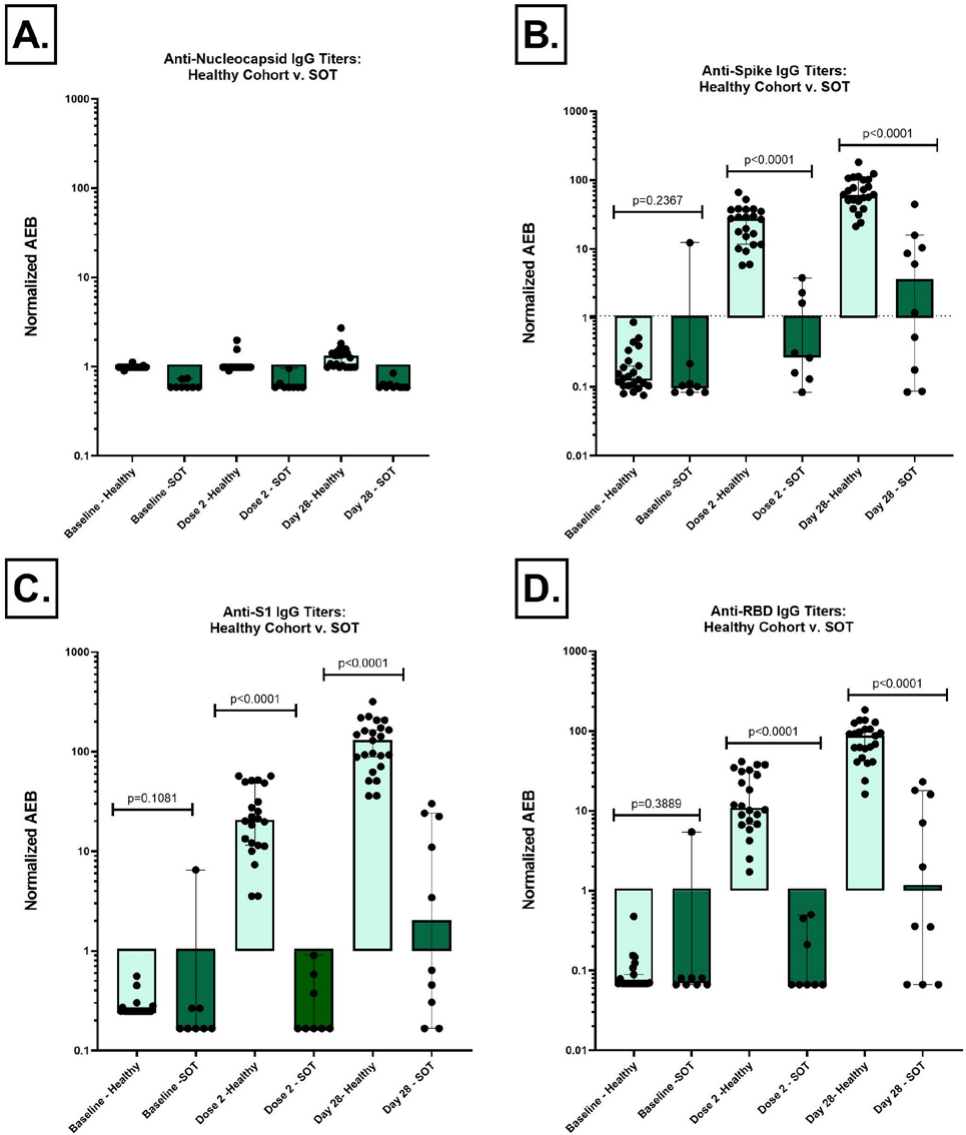

Black error bars denote median and 95% CI. The dotted line on panel B denotes an internally validated cutoff of 1.07; anti-S IgG titers greater than 1.07 denote a positive response.

Figure 3: Time from Transplant v. anti-S IgG Titer

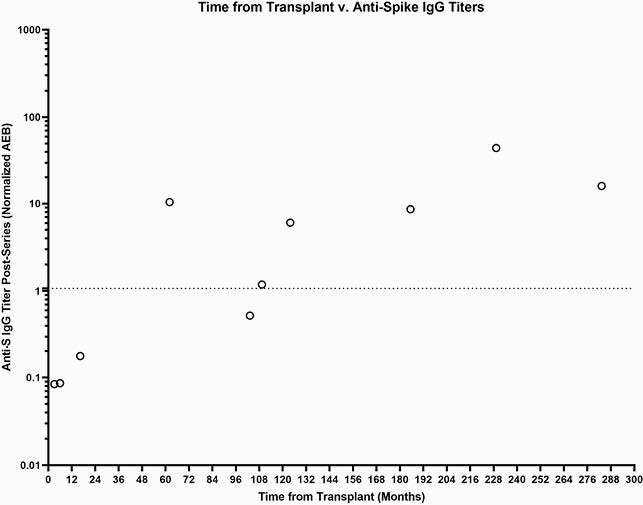

SOT recipients further out from transplant tend to have a higher anti-S IgG response. The dotted line denotes an internally validated cutoff, with anti-S IgG titers greater than 1.07 indicating a positive response.

**Conclusion:**

SOT recipients had a significantly decreased humoral response to mRNA COVID-19 vaccines compared to the healthy cohort, with those further out from transplant more likely to respond. Further research is needed to evaluate T-cell responses and clinical efficacy to maximize the SARS-CoV-2 vaccine response among SOT recipients.

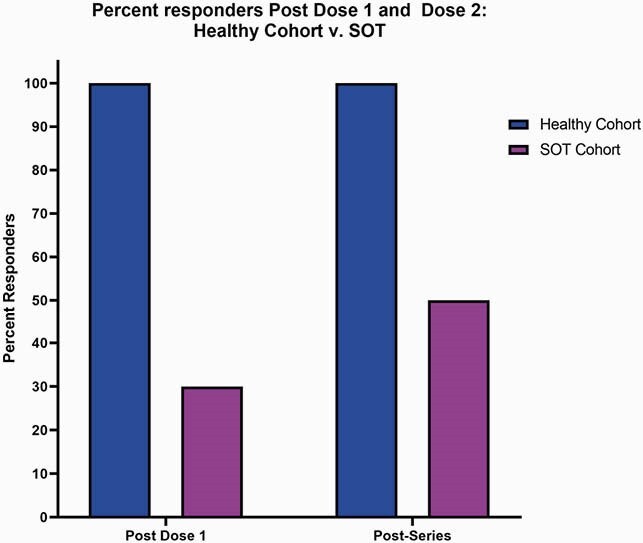

**Disclosures:**

**Ann E. Woolley, MD, MPH**, **COVAX** (Consultant) **David Walt, PhD**, **Quanterix Corporation** (Board Member, Shareholder)

